# Clinical, Bacterial, and Antibiogram Profiles of Infections Among Type 2 Diabetes Mellitus Patients in a Tertiary Care Center

**DOI:** 10.7759/cureus.115889

**Published:** 2026-09-07

**Authors:** Prakash, Hina Mehra, Jayasree Sivadasan

**Affiliations:** 1 Department of General Medicine, Apollo Hospitals, Bengaluru, IND

**Keywords:** antibiogram, antimicrobial resistance, bacterial infections, multidrug resistance, type 2 diabetes mellitus

## Abstract

Background

Patients with type 2 diabetes mellitus (T2DM) have an increased susceptibility to infection because of hyperglycemia-related immune dysfunction, vascular impairment, and associated comorbidities. Characterization of bacterial isolates and their antimicrobial susceptibility patterns is essential for selecting appropriate empirical therapy and limiting antimicrobial resistance.

Methodology

This hospital-based, cross-sectional study included 150 adults with T2DM and culture-positive infections admitted to a tertiary care hospital in Bengaluru, India, between April 2023 and November 2024. Clinical and microbiological characteristics, HbA1c levels, intensive care unit (ICU) admission, and in-hospital mortality were assessed. Bacterial identification, antimicrobial susceptibility testing, and extended-spectrum beta-lactamase (ESBL) assessment were performed by the hospital microbiology laboratory as part of routine clinical care, with susceptibility results reported according to the applicable Clinical and Laboratory Standards Institute criteria.

Results

Urinary tract infection was the most frequent infection, affecting 55/150 patients (36.67%). *Escherichia coli* was the predominant isolate (54/150 (36.00%)), followed by *Klebsiella* spp. (26/150 (17.33%)) and *Pseudomonas* spp. (24/150 (16.00%)). Multidrug-resistant (MDR) organisms were identified in 53/150 patients (35.33%), while ESBL production was recorded in 50/118 isolates with an evaluable ESBL result (42.37%); 32 isolates were classified as not applicable. Resistance was highest to meropenem (64/150 (42.67%)), followed by amoxicillin (60/150 (40.00%)) and ciprofloxacin (58/150 (38.67%)). Although glycemic control status differed between the MDR and non-MDR groups (p = 0.033), uncontrolled glycemia was less frequent in the MDR group; therefore, this finding does not establish poor glycemic control as a predictor of MDR infection. MDR infections were associated with higher ICU admission rates (26/53 (49.06%) versus 13/97 (13.40%); p < 0.001) and in-hospital mortality (9/53 (16.98%) versus 5/97 (5.15%); p = 0.017).

Conclusions

MDR bacterial infections were common among hospitalized patients with T2DM and were associated with higher rates of ICU admission and in-hospital mortality. Institution-specific antibiograms, prompt culture-guided therapy, and strengthened antimicrobial stewardship are needed given the observed resistance, including resistance to meropenem.

## Introduction

Diabetes mellitus is a chronic metabolic disease characterized by hyperglycemia resulting from impaired insulin secretion, impaired insulin action, or both. Type 2 diabetes mellitus (T2DM), its most common form, is associated with metabolic, vascular, neurological, renal, and immune complications that contribute substantially to morbidity, healthcare utilization, and mortality [1]. In 2021, approximately 537 million adults worldwide were living with diabetes, and this number is projected to reach 783 million by 2045. India carries a considerable share of this burden, with more than 77 million adults affected in 2019 [2].

Patients with T2DM have an increased susceptibility to infection. Glycosuria, autonomic neuropathy, incomplete bladder emptying, and impaired host defenses increase the risk of recurrent and complicated urinary tract infections (UTIs) [3]. In Indian hospital settings, Gram-negative organisms, particularly *Escherichia coli*, are prominent causes of UTIs among patients with diabetes, and resistance to commonly used antimicrobial agents can complicate empirical treatment [4]. The infectious burden is not restricted to the urinary tract; hospitalized patients with T2DM may also develop lower respiratory tract infections, skin and soft-tissue infections, abdominal infections, bloodstream infections, and sepsis [5]. Diabetic foot infections represent an important component of this burden because neuropathy, vascular insufficiency, delayed wound recognition, and impaired healing can permit infection progression [6].

Antimicrobial resistance further complicates the management of infections in patients with diabetes. Studies of bacteriuria in diabetic populations have demonstrated considerable bacterial diversity and resistance to commonly prescribed antimicrobial agents [7]. Multidrug-resistant (MDR) organisms are particularly concerning because they restrict therapeutic options, increase the risk of inappropriate initial therapy, and may contribute to prolonged hospitalization and greater treatment costs [8]. The distribution of pathogens and their susceptibility patterns varies across patient populations, healthcare institutions, geographic regions, and patterns of antimicrobial exposure [9]. Antibiograms derived from other settings may therefore not reliably represent local resistance patterns.

Glycemic status may also influence susceptibility to infection and its clinical course. Population-based evidence indicates that poor glycemic control is associated with an increased risk of infection, although the strength of this association varies according to infection type, diabetes duration, comorbidities, and other patient characteristics [10]. Hyperglycemia can impair neutrophil chemotaxis, phagocytosis, intracellular bacterial killing, cytokine production, and cell-mediated immunity. Vascular insufficiency may further reduce tissue perfusion, oxygen delivery, immune cell access, and antimicrobial penetration, thereby contributing to persistent or recurrent infection [11]. People with diabetes consequently experience an increased risk of several infectious diseases, emphasizing the importance of early recognition, microbiological evaluation, and timely treatment [12].

Institution-specific microbiological surveillance is essential because resistance profiles may differ according to specimen type, infection site, patient population, and local antimicrobial use. Substantial resistance has also been documented among bacterial isolates recovered from wound and pus specimens in tertiary care hospitals [13]. Identification of locally prevalent pathogens and their antimicrobial susceptibility patterns is therefore necessary to guide empirical therapy, support antimicrobial stewardship, and reduce treatment failure.

Objectives of the study

Local evidence describing the spectrum of bacterial infections and antimicrobial resistance among hospitalized patients with T2DM remains limited. This study therefore aimed to characterize these patterns and evaluate prespecified associations involving glycemic control and clinical outcomes. The primary objective was to describe the anatomical sites of infection, bacterial isolate distribution, and antimicrobial susceptibility patterns among hospitalized patients with T2DM and culture-confirmed bacterial infections. The exploratory secondary objectives were to assess the associations of HbA1c-based glycemic control status with bacterial isolate distribution, MDR status, and extended-spectrum beta-lactamase (ESBL) status, and to assess the associations of MDR status with intensive care unit (ICU) admission and all-cause in-hospital mortality.

## Materials and methods

Study design and setting

This hospital-based, cross-sectional, observational study was conducted in the Department of Internal Medicine and Allied Specialties at Apollo Hospitals, Bengaluru, a tertiary care hospital with established microbiological diagnostic facilities. The study was conducted over 18 months, from April 2023 to November 2024. Ethical approval was obtained from the Institutional Ethics Committee before commencement of the study.

Study population and sampling

A total of 150 adults with previously diagnosed T2DM and culture-positive acute bacterial infections were recruited consecutively. Patients were eligible if they were aged ≥18 years, had an infection of less than three weeks’ duration, and had been hospitalized for less than 48 hours at enrollment. Patients with culture-negative infections, infections developing ≥48 hours after admission, active tuberculosis, HIV/AIDS, long-term immunosuppressive therapy, or absence of informed consent were excluded. The study assessed infection sites, bacterial isolates, antimicrobial susceptibility patterns, ICU admission, and all-cause in-hospital mortality. Reinfection, recurrence, readmission, and post-discharge outcomes were not evaluated. The sample size was calculated using the prevalence of different infections among hospitalized patients with diabetes [5]. The formula used was:

$n=Z^2 p q / d^2$

where n was the required sample size, Z was 1.96 at a 95% confidence level, p was the anticipated prevalence, q=1−p, and d was the absolute precision of 5%. The highest required sample size was obtained using the reported prevalence of skin and soft-tissue infection of 10.5%: n = (1.96)² × 0.105 × 0.895/(0.05)² = 144.4, which was rounded to 145. A final sample of 150 participants was included in the study.

Data collection

A structured case-record form was used to collect data. The recorded variables included age, sex, duration of diabetes, comorbid conditions, presenting symptoms, anatomical site of infection, and relevant clinical characteristics. Laboratory parameters included HbA1c, fasting blood glucose, and postprandial blood glucose. HbA1c was used as the principal measure of glycemic control status. Controlled glycemia was defined as HbA1c ≤7.0%, and uncontrolled glycemia was defined as HbA1c >7.0%. The microbiological variables included the bacterial isolate, antimicrobial susceptibility pattern, MDR status, and ESBL production. ICU admission and in-hospital mortality were recorded as clinical outcomes. Prior antibiotic exposure before hospital admission was not evaluated as a predefined study variable.

Microbiological evaluation and study outcomes

Appropriate clinical specimens, including blood, urine, sputum, wound swabs, abscess material, endotracheal aspirates, and other body fluid samples, were collected according to the suspected anatomical site of infection and submitted to the hospital microbiology laboratory. Wherever clinically feasible, specimens were collected before initiating empirical intravenous antibiotic therapy. Patients presenting with fever, sepsis, purulent discharge, or other clinical evidence of infection received empirical intravenous antibiotics after the appropriate blood, urine, or site-specific specimens had been obtained. Antibiotic treatment was subsequently modified according to the organism identification and antimicrobial susceptibility results reported by the microbiology laboratory. The study investigators did not perform the microbiological testing independently. Bacterial identification, antimicrobial susceptibility testing, and ESBL assessment were conducted as part of routine clinical care by the hospital microbiology laboratory using the manual and automated procedures operational during the study period. Susceptibility results were interpreted according to the applicable Clinical and Laboratory Standards Institute criteria. The investigators extracted the organism identification, susceptibility classification, and ESBL result from the finalized microbiology reports. Only organism-antibiotic combinations documented in these reports were included in the analysis. Intermediate or unreported results were not reclassified as resistant.

An isolate was classified as MDR when it demonstrated acquired non-susceptibility to at least one antimicrobial agent in three or more antimicrobial categories on the complete organism-specific susceptibility panel. ESBL status was recorded only for applicable Enterobacterales isolates when the finalized laboratory report documented the result. Organisms for which ESBL testing was not applicable or not reported were categorized separately and were not considered ESBL-negative. The study variables included demographic characteristics, duration of diabetes, HbA1c-based glycemic control status, infection site, bacterial isolate, antimicrobial susceptibility pattern, MDR status, and ESBL production. The primary analysis described the clinical spectrum of culture-positive infections, bacterial isolate distribution, and recorded antibiogram patterns among hospitalized patients with T2DM. ICU admission and all-cause in-hospital mortality during the index hospitalization were assessed as clinical outcomes. Exploratory secondary analyses examined the associations of glycemic-control status with bacterial isolate distribution, MDR status, and ESBL status, and the associations of MDR status with ICU admission and mortality. Reinfection, recurrence, readmission, and post-discharge outcomes were not evaluated. These associations were interpreted as exploratory and not causal because potential confounders, including previous antimicrobial exposure, recent hospitalization, infection severity, invasive device exposure, and detailed comorbidity burden, were not consistently collected.

Statistical analysis

Categorical variables were summarized as frequencies and percentages. Pearson’s chi-square test was used to assess the associations between infection site and bacterial isolate distribution, glycemic control status and bacterial isolate distribution, glycemic control status and MDR status, glycemic control status and ESBL status, MDR status and ICU admission, and MDR status and in-hospital mortality. No multivariable analysis was performed because prior antimicrobial exposure, recent hospitalization, invasive device exposure, infection severity, and detailed comorbidity burden were not consistently collected. No correction for multiple comparisons was applied; therefore, the secondary analyses were considered exploratory, and statistically significant results were not interpreted as independent or causal associations. Completeness of the variables included in the primary and secondary analyses was assessed before analysis. No missing observations were identified for age, sex, duration of diabetes, HbA1c-based glycemic control status, infection site, bacterial isolate, MDR status, ICU admission, in-hospital mortality, or the antimicrobial susceptibility variables summarized in the analysis; therefore, no imputation of missing values was required. ESBL status was evaluable in 118/150 (78.67%) isolates; the remaining 32/150 (21.33%) isolates were classified as not applicable/not reported according to the finalized microbiology report and were retained as a separate category rather than treated as ESBL-negative. Analyses were performed using the available recorded data for each predefined variable.

Ethical considerations

Ethical approval was obtained from the Institutional Ethics Committee of Apollo Hospitals, Bengaluru (approval number: AHB/IEC/DNB/GENERAL MEDICINE/070/2023).

## Results

Demographic and glycemic characteristics

There were 150 patients with T2DM and culture-positive infections. A slight female predominance was found, and patients aged over 60 years formed the largest age group. A diabetes duration of 11-20 years was most common among the participants. Based on the predefined HbA1c threshold, 119/150 (79.33%) patients had uncontrolled glycemia (HbA1c >7.0%), whereas 31/150 (20.67%) had controlled glycemia (HbA1c ≤7.0%). Demographic and glycemic characteristics are shown in Table 1.

**Table 1:** Demographic and glycemic characteristics of the study population. Data are presented as frequency (n), total number of patients (N), and percentage (%). No inferential statistical analysis was performed. Controlled glycemia: HbA1c ≤7.0%; uncontrolled glycemia: HbA1c >7.0%. HbA1c: glycated hemoglobin

Parameter	Category	n	N	%
Age (years)	>60	60	150	40.00
	30–60	58	150	38.67
	18–30	32	150	21.33
Sex	Female	80	150	53.33
	Male	70	150	46.67
Duration of diabetes	11–20 years	81	150	54.00
	≤10 years	65	150	43.33
	>20 years	4	150	2.67
Glycemic control based on HbA1c	Uncontrolled	119	150	79.33
	Controlled	31	150	20.67

There were no missing observations for the principal demographic, glycemic, infection site, bacterial isolate, MDR status, ICU admission, mortality, or summarized antimicrobial susceptibility variables among the 150 participants. ESBL status was evaluable in 118/150 (78.67%) isolates; 32/150 (21.33%) isolates were categorized as not applicable/not reported and were analyzed separately.

Distribution of infection sites and bacterial isolates

UTI was the most common infection (55/150 (36.67%)), followed by skin and soft-tissue infection (33/150 (22.00%)), lower respiratory tract infection (31/150 (20.67%)), abdominal infection (20/150 (13.33%)), bloodstream infection/sepsis (8/150 (5.33%)), and other infections (3/150 (2.00%)). *E. coli* was the leading isolate in UTIs (22/55 (40.00%)), abdominal infections (11/20 (55.00%)), lower respiratory tract infections (8/31 (25.81%)), bloodstream infection/sepsis (4/8 (50.00%)), and other infections (2/3 (66.67%)). *E. coli* and *Klebsiella* spp. were equally frequent in skin and soft-tissue infections (7/33 (21.21%) each). The distribution of bacterial isolates did not differ significantly among infection sites (χ² = 31.02, df = 30, p = 0.414). The distribution is presented in Table 2.

**Table 2:** Distribution of infection sites and leading bacterial isolates. Data are presented as frequency (n), total number of patients (N), site total, and percentage (%), as applicable. Pearson’s chi-square test was performed to assess the association between infection site and bacterial isolate distribution. Statistical significance was defined as p < 0.05. Other infections comprised bacterial meningitis (n = 1), sinusitis (n = 1), and endophthalmitis (n = 1). Within this category, *E. coli* was isolated in two cases and *Pseudomonas* spp. in one case. *E. coli*: *Escherichia coli*; χ²: chi-square statistic; df: degrees of freedom

Site of infection	Site n	Total N	Site %	Leading bacterial isolate	Isolate n	Site total	Isolate %
Urinary tract infection	55	150	36.67	E. coli	22	55	40.00
Skin and soft-tissue infection	33	150	22.00	E. coli	7	33	21.21
Skin and soft-tissue infection	33	150	22.00	*Klebsiella* species	7	33	21.21
Lower respiratory tract infection	31	150	20.67	E. coli	8	31	25.81
Abdominal infection	20	150	13.33	E. coli	11	20	55.00
Sepsis	8	150	5.33	E. coli	4	8	50.00
Other infections	3	150	2.00	E. coli	2	3	66.67

Antimicrobial resistance profile

MDR organisms were identified in 53/150 (35.33%) patients. ESBL production was recorded in 50/118 (42.37%) isolates with an evaluable ESBL result; the remaining 32 isolates were classified as not applicable and excluded from the ESBL denominator. Meropenem demonstrated the highest resistance rate (64/150 (42.67%)), followed by amoxicillin (60/150 (40.00%)), ciprofloxacin (58/150 (38.67%)), piperacillin-tazobactam (54/150 (36.00%)), and ceftriaxone (54/150 (36.00%)). The complete antimicrobial susceptibility profile is presented in Table 3.

**Table 3:** Antimicrobial sensitivity and resistance profiles. The five antibiotics shown represent the summarized antibiogram, whereas MDR status was determined using the complete organism-specific antimicrobial susceptibility panel. Data are presented as frequency (n), total number of isolates tested (N), and percentage (%). Ceftriaxone resistance was observed in 54/150 (36.00%) isolates. No inferential statistical analysis was performed. AST: antimicrobial susceptibility testing; MDR: multidrug-resistant

Antibiotic	Sensitive n	N tested	Sensitive %	Resistant n	N tested	Resistant %
Amoxicillin	90	150	60.00	60	150	40.00
Ciprofloxacin	92	150	61.33	58	150	38.67
Meropenem	86	150	57.33	64	150	42.67
Piperacillin-tazobactam	96	150	64.00	54	150	36.00
Ceftriaxone	96	150	64.00	54	150	36.00

The recorded susceptibility results varied across bacterial groups. Because organism-specific testing panels, interpretive breakpoints, and intrinsic resistance profiles differed, pooled resistance frequencies were considered descriptive and were not interpreted as directly comparable antibiotic-specific resistance rates. Among *E. coli* isolates, meropenem resistance was recorded in 26/54 (48.15%). *Klebsiella* spp. showed the highest recorded resistance to ciprofloxacin (13/26 (50.00%)). Among *Pseudomonas* spp., resistance to meropenem and ciprofloxacin was recorded in 7/24 (29.17%) isolates each, while piperacillin-tazobactam resistance was recorded in 6/24 (25.00%) isolates. The organism-specific findings are presented in Table 4.

**Table 4:** Organism-specific antimicrobial resistance profiles. Data are presented as resistant isolates/total isolates tested within each bacterial group (%). This was a descriptive analysis; no inferential comparisons among bacterial groups were performed. *E. coli*: *Escherichia coli*

Antibiotic	*E. coli* (n = 54), resistant n/N (%)	*Klebsiella* spp. (n = 26), resistant n/N (%)	*Pseudomonas* spp. (n = 24), resistant n/N (%)
Amoxicillin	21/54 (38.89)	10/26 (38.46)	13/24 (54.17)
Ciprofloxacin	20/54 (37.04)	13/26 (50.00)	7/24 (29.17)
Meropenem	26/54 (48.15)	12/26 (46.15)	7/24 (29.17)
Piperacillin-tazobactam	20/54 (37.04)	11/26 (42.31)	6/24 (25.00)
Ceftriaxone	17/54 (31.48)	9/26 (34.62)	10/24 (41.67)

Association of glycemic control with antimicrobial resistance

Pearson’s chi-square test showed a statistically significant association between MDR status and glycemic control (χ² = 4.53, df = 1, p = 0.033). Of the 119 patients with uncontrolled glycemia, 37 (31.09%) had MDR infections and 82 (68.91%) had non-MDR infections. Of the 31 patients with controlled glycemia, 16 (51.61%) had MDR infections, and 15 (48.39%) had non-MDR infections. Thus, the 37 patients represented the MDR subgroup within the total of 119 patients with uncontrolled glycemia. No statistically significant association was observed between glycemic control status and ESBL status (χ² = 1.72, df = 2, p = 0.423) or bacterial isolate distribution (χ² = 7.25, df = 6, p = 0.298).

Association of multidrug-resistant infections with clinical outcomes

MDR organisms were identified in 53/150 (35.33%) patients. Overall, 39/150 (26.00%) patients required ICU admission and 14/150 (9.33%) died during hospitalization. ICU admission was significantly more frequent among patients with MDR infections than among those with non-MDR infections (26/53 (49.06%) versus 13/97 (13.40%); χ² = 22.65, df = 1, p < 0.001). In-hospital mortality was also significantly higher in the MDR group (9/53 (16.98%) versus 5/97 (5.15%); χ² = 5.66, df = 1, p = 0.017). These findings represent unadjusted associations and do not establish that MDR infection independently caused ICU admission or mortality. The associations of MDR status with glycemic control and clinical outcomes are summarized in Table 5.

**Table 5:** Associations of MDR status with glycemic control and clinical outcomes. Data are presented as n/category total (%). Pearson’s chi-square test was used for all comparisons, with the chi-square statistic (χ²), degrees of freedom (df), and corresponding p-value reported. Statistical significance was defined as p < 0.05. For the ESBL analysis, isolates were classified as ESBL-positive (n = 50), ESBL-negative (n = 68), or not applicable/not reported (n = 32); all three categories were included in the reported chi-square analysis. ESBL: extended-spectrum beta-lactamase; ICU: intensive care unit; MDR: multidrug-resistant

Comparison	Category 1, n/N (%)	Category 2, n/N (%)	χ²	df	P-value
MDR status versus glycemic control	Controlled: MDR 16/31 (51.61), non-MDR 15/31 (48.39)	Uncontrolled: MDR 37/119 (31.09), non-MDR 82/119 (68.91)	4.53	1	0.033
ESBL status versus glycemic control	Controlled: negative 15/68 (22.06), not applicable 4/32 (12.50), positive 12/50 (24.00)	Uncontrolled: negative 53/68 (77.94), not applicable 28/32 (87.50), positive 38/50 (76.00)	1.72	2	0.423
Bacterial isolate distribution versus glycemic control	Controlled: 31/150 (20.67) across seven isolate categories	Uncontrolled: 119/150 (79.33) across seven isolate categories	7.25	6	0.298
MDR status versus ICU admission	MDR: 26/53 (49.06)	Non-MDR: 13/97 (13.40)	22.65	1	<0.001
MDR status versus in-hospital mortality	MDR: 9/53 (16.98)	Non-MDR: 5/97 (5.15)	5.66	1	0.017

## Discussion

This study described the infection sites, bacterial isolates, and antimicrobial susceptibility patterns among 150 hospitalized patients with T2DM and culture-confirmed infections. The exploratory secondary analyses evaluated the associations of glycemic control status with bacterial isolate distribution, MDR status, and ESBL status, and the associations of MDR status with ICU admission and in-hospital mortality. UTI was the most common infection (55/150 (36.67%)), and *E. coli* was the predominant bacterial isolate (54/150 (36.00%)). MDR organisms were identified in 53/150 (35.33%) patients, while ESBL production was recorded in 50/118 (42.37%) isolates with an evaluable result; 32 isolates were classified as not applicable. The highest recorded overall resistance was to meropenem (64/150 (42.67%)), followed by amoxicillin (60/150 (40.00%)) and ciprofloxacin (58/150 (38.67%)). Glycemic control status differed significantly between the MDR and non-MDR groups (p = 0.033); however, uncontrolled glycemia was less frequent among patients with MDR infections, and this finding should not be interpreted as evidence that poor glycemic control increased the likelihood of MDR infection. MDR infections were significantly associated with higher ICU admission (26/53 (49.06%) versus 13/97 (13.40%); p < 0.001) and in-hospital mortality (9/53 (16.98%) versus 5/97 (5.15%); p = 0.017). These were exploratory, unadjusted associations and do not establish MDR infection as an independent predictor or cause of ICU admission or mortality.

UTI was the most frequently observed infection, consistent with studies reporting a high prevalence of Gram-negative, MDR uropathogens among patients with diabetes [8,9]. Persistent bacterial colonization of the urinary tract may be associated with glycosuria, impaired bladder emptying, autonomic dysfunction, and diminished local immunity [9]. The bacterial distribution observed in this study was also similar to that reported in tertiary care settings. Studies of pus and wound isolates have frequently identified Gram-negative bacteria and considerable resistance to commonly used antibiotics [13]. Diabetic foot infections have also demonstrated diverse bacterial flora, including Gram-positive and Gram-negative organisms [14]. *E. coli* has similarly been identified as the predominant isolate in hospital-based studies of UTIs among patients with T2DM [15]. The predominance of *E. coli* in the present study is consistent with the reported distribution among patients with diabetes, although bacterial patterns may differ according to infection site, geographic location, referral patterns, and previous antibiotic exposure.

The present study identified a substantial descriptive burden of MDR organisms and ESBL production. ESBL production was recorded in 50/118 (42.37%) isolates with an evaluable result. The evaluable denominator of 118 was used instead of the total study population because ESBL testing was classified as not applicable for 32 isolates. Previous studies have reported ESBL-producing Gram-negative organisms and methicillin-resistant *Staphylococcus aureus* in diabetic foot infections [16], indicating that resistant pathogens are present in complicated diabetic foot infections. Reduced susceptibility to commonly used antimicrobial agents has also been reported among bacterial isolates obtained from diabetic foot ulcers [17]. A similar study of UTIs among patients with diabetes identified ESBL-producing Gram-negative organisms and resistance to several antibiotic classes [18]. Antimicrobial resistance has also been observed among wound isolates from tertiary care hospitals, with patterns comparable to those reported for other specimen types [19]. Prospective studies of postoperative wound infections have also reported substantial antimicrobial resistance among bacterial isolates, emphasizing the need for continuous local surveillance [20]. The recorded overall meropenem resistance rate of 64/150 (42.67%) is clinically concerning but should be interpreted cautiously because antimicrobial panels, clinical applicability, and intrinsic resistance differ among bacterial species. Previous antimicrobial exposure, referral of complicated cases, selection pressure, or acquisition of resistance mechanisms could have contributed to the observed resistance; however, these explanations remain hypothetical because prior antibiotic exposure and molecular resistance mechanisms were not evaluated. The present findings do not support recommending a single empirical antibiotic regimen because susceptibility testing panels and intrinsic resistance profiles differed among organisms, while infection site, infection severity, previous antibiotic exposure, renal function, and recent healthcare exposure were not incorporated into the analysis. Empirical treatment should therefore be selected according to the suspected infection site, clinical severity, patient-specific risk factors, and the current institution-specific, organism-stratified antibiogram, followed by prompt modification or de-escalation when culture and susceptibility results become available. The pooled meropenem resistance frequency should not be used alone to determine empirical therapy.

Glycemic control status differed significantly according to MDR status (χ² = 4.53, df = 1, p = 0.033), but the direction of the association was unexpected. MDR infection was identified in 37/119 (31.09%) patients with uncontrolled glycemia compared with 16/31 (51.61%) patients with controlled glycemia. This finding should not be interpreted as indicating that uncontrolled glycemia protects against MDR infection. The inverse pattern may reflect residual confounding by factors not included in the analysis, such as previous antibiotic exposure, recent hospitalization, comorbidity burden, diabetes duration, infection site, and infection severity. Selection bias may also have contributed because the study was restricted to hospitalized patients with culture-positive infections, excluding patients with culture-negative infections and those managed outside the hospital. In addition, the relatively small controlled glycemia group (n = 31), use of a single dichotomized HbA1c measure, absence of multivariable adjustment, and multiple unadjusted comparisons may have affected the stability and interpretation of the association. No significant association was identified between glycemic control status and ESBL status (χ² = 1.72, df = 2, p = 0.423) or bacterial isolate distribution (χ² = 7.25, df = 6, p = 0.298).

The current findings therefore neither establish poor glycemic control as a predictor of MDR infection nor demonstrate a protective effect of uncontrolled glycemia. Prospective studies with larger samples and multivariable adjustment are required to determine whether glycemic control is independently associated with MDR infection. Recent evidence further supports the clinical importance of MDR surveillance and antimicrobial stewardship in patients with diabetes. Yovera-Aldana et al. reported that MDR bacteria were independently associated with hospitalization exceeding 14 days among patients with diabetic foot infection [21]. A recent study reported that 59.0% of *E. coli* isolates obtained from patients with diabetes and complicated UTIs were MDR, supporting risk-stratified empirical therapy based on local resistance patterns [22]. These findings support culture-guided treatment, timely de-escalation, and institution-specific stewardship protocols. The associations between MDR status, ICU admission, and mortality in the present study may have been influenced by infection severity, prior antimicrobial exposure, recent hospitalization, comorbidities, and other unmeasured factors. MDR status should therefore not be considered an independent risk factor for poor outcomes based on this cross-sectional, unadjusted analysis.

Strengths, limitations, and future directions

The strengths of this study include consecutive recruitment of patients with culture-confirmed infections, assessment of multiple infection sites, and evaluation of bacterial isolates, antimicrobial susceptibility, MDR and ESBL status, ICU admission, and in-hospital mortality. The unexpected MDR-glycemic-control pattern and high recorded meropenem resistance were interpreted cautiously. The single-center, cross-sectional design, inclusion of only hospitalized patients, and absence of a non-diabetic comparator limit generalizability and causal interpretation. Prior antibiotic exposure, recent hospitalization, invasive device exposure, comorbidities, and infection severity were not systematically recorded or adjusted for. Cultures obtained from non-sterile sites may have represented colonization or contamination.

Microbiological testing was undertaken as part of routine clinical care, and finalized laboratory reports were used for analysis. The precise culture conditions, identification and antimicrobial susceptibility testing platforms, Clinical and Laboratory Standards Institute edition and breakpoints, quality control strains, and ESBL confirmation procedures were not captured in the research dataset and could not be reconstructed reliably from the finalized laboratory reports. This reporting limitation restricts exact microbiological reproducibility and should be considered when interpreting the resistance estimates. Differences in organism-specific antimicrobial panels, the small controlled glycemia subgroup, dichotomized HbA1c, and multiple unadjusted comparisons further restrict interpretation. The findings are therefore descriptive and hypothesis-generating. Future multicenter prospective studies should include comparator groups, standardized infection severity criteria, complete microbiological protocols, organism-specific antibiograms, systematic assessment of potential confounders, and multivariable analyses.

## Conclusions

In this cross-sectional study of 150 hospitalized patients with T2DM and culture-confirmed bacterial infections, UTI was the most frequent infection, and *E. coli* was the predominant isolate. MDR organisms were identified in 53/150 (35.33%) infections, while ESBL production was recorded in 50/118 (42.37%) isolates with an evaluable result; 32 isolates were classified as not applicable. ICU admission and in-hospital mortality were more frequent in the MDR group, but these unadjusted findings do not establish independent or causal relationships. The unexpected inverse distribution between uncontrolled glycemia and MDR infection may reflect residual confounding, selection bias, subgroup imbalance, or multiple comparisons and should not be interpreted as a protective effect. Pooled resistance estimates should be interpreted cautiously because organism-specific panels, breakpoints, and intrinsic resistance profiles differed. The findings are descriptive and hypothesis-generating and support culture-guided treatment and organism-specific institutional surveillance.
